# Effects of Voluntary Running Wheel Activity and Hypertension on the Brain of Female Spontaneously Hypertensive Rats (SHRs)

**DOI:** 10.3390/ijms27073182

**Published:** 2026-03-31

**Authors:** Tsunehisa Sato, Rolf Schreckenberg, Klaus-Dieter Schlüter

**Affiliations:** 1 Department of Anesthesiology and Intensive Care, Hamamatsu University of Medicine, Hamamatsu 431-3192, Japan; sato_tsune@outlook.ij; 2 Physiologisches Institut, Justus-Liebig-Universität, 35392 Giessen, Germany; rolf.schreckenberg@physiologie.med.uni.giessen.de

**Keywords:** vascular dementia, uncoupling proteins, oxidative stress

## Abstract

Low physical activity is a common risk factor for hypertension and dementia. We investigated whether voluntary running wheel activity (VRWA) ameliorates the effects of hypertension in the brain. Forty-six six-week-old female spontaneously hypertensive rats were randomly selected in a sedentary control group (SHR-S; *n* = 21) or had access to running wheels during their active nighttime (SHR-R; *n* = 15). Age-matched normotensive Wistar rats served as controls (WIS; *n* = 10). Animals were sacrificed after six months. The cortex, medulla oblongata, and olfactory bulb were prepared. Oxidative stress was analyzed by DHE staining, protein expression by Western blots, mRNA expression by qRT-PCR and blood pressure by a tail-cuff method. VRWA reduced heart rates but not blood pressure. All SHRs displayed a strong reduction of *Ucp2* brain expression in a blood-pressure-dependent way. VRWA did not improve the expression of *Ucp2* but increased the expression of *Cat* and reduced oxidative stress in the cortex. Hypertension increased the expression of *Ren* in the medulla oblongata without any effect of VRWA on this parameter. VRWA generally affected mRNA expression stronger in the cortex than in the other parts of the brain. In conclusion, high physical activity ameliorated oxidative stress in the cortex in a blood-pressure-independent way.

## 1. Introduction

With an increasingly aged population, hypertension-dependent end-organ diseases drive the high socio-economic burden of the public health systems. Therapies and patients’ care are challenging. Hypertension is associated with long-term complications such as stroke, heart failure, or chronic kidney disease. Moreover, hypertension and more specific midlife hypertension are also associated with vascular dementia or vascular cognitive impairment and Alzheimer’s disease [1,2,3,4,5,6]. The interaction between hypertension, ageing, and dementia is complex but a coupling between vascular damage as it occurs under hypertensive conditions and dementia is likely [4]. The molecular mechanisms triggering these adverse events are poorly understood. For example, in an epidemiological study it was found that older females had a higher risk of developing Alzheimer’s disease compared with age-matched males, although the degree of hypertension and the absolute blood pressure was comparable between females and males [7]. There are various limitations among such studies but, overall, in female hypertensive patients blood pressure raises later than in male patients, though female patients have a comparable risk to develop dementia in the presence of hypertension.

In general, risk factors leading to hypertension and dementia are either non-modifiable risk factors, such as genetic predisposition, or modifiable risk factors such as physical inactivity. Indeed, several preclinical studies with rodents showed a protective role of physical activity against the onset of Alzheimer’s disease [8,9]. Mechanistically, exercise may improve the blood–brain barrier function in hypertensives. Loss in blood–brain barrier function contributes to oxidative stress [5,10]. In our study, we used the advantage of an established hypertension model, namely spontaneously hypertensive rats (SHRs), to study the relationship between hypertension and dementia, as SHR showed impaired memory by ageing [11]. Female SHRs have a higher intrinsic motivation to perform voluntary running wheel activity (VRWA) than their male counterparts [12]. Furthermore, hypertensive females require a specific view in the context of dementia [7]. VRWA allows the animals to exercise in a stress-free way. Rats can run during the night in their natural active phase and at a self-selected intensity. Monitoring of the VRWA allows investigation of load-dependent effects as well, because not all rats use the wheels for the same time. This type of physical activity does not lead to alterations in blood pressure [13,14,15]. Therefore, any effects of voluntary running wheel activity on the brain are exercise dependent but not blood-pressure dependent. In summary, we consider the female, adult SHR to be a suitable model by which to investigate the effects of hypertension on the brain and the potential impact of higher activity.

The brain is a highly differentiated organ in which different areas have specific functions. Here we analyzed three different parts of the brain. The medulla oblongata was chosen because it is centrally involved in blood pressure regulation. The olfactory bulb was chosen because it is the main distance sense in rats. It is therefore important in terms of the influence of external stress on circulation. The cortex was chosen because it is the main part of the brain responsible for cognition and memory.

As in other organs and diseases, oxidative stress also impairs brain function and is likely to participate in the progression of dementia [16,17]. Oxidative stress reduces the conductivity of potassium channels, thereby altering the excitability of neurons [18]. On the other hand, the brain is very sensitive against oxidative stress produced in mitochondria due to its high energy demand. Indeed, mitochondrial dysfunction is associated with neurodegenerative diseases [19,20]. Uncoupling proteins (UCPs) are located at the inner mitochondrial membrane. They trigger mitochondrial-derived oxidative stress and fuel metabolism in neurons and other cells [21,22,23]. The brain is unique in the expression profile of UCP isoforms. It expresses four of the five known and different UCPs, namely the isoforms 2, 3, 4, and 5. It has been shown, at least for UCP4, that genetic variants are associated with Alzheimer’s disease [24]. UCP2 is highly expressed in the brain of mice and knockout of UCP2 triggers neurological defects such as anxiety-like behavior, depression-like behavior, and mental illness in mice [25,26,27]. Whether UCP2 plays a similar role in other species, such as rats, is currently not known. Here, we focused on the effect of hypertension on the expression of uncoupling proteins and their interaction with physical activity.

In summary, our study is aimed at identifying molecular targets that link hypertension to Alzheimer’s disease and those that can be affected by physical activity in female hypertensive rats.

## 2. Results

### 2.1. Effect of Voluntary Running Wheel Activity on Heart Rate and Blood Pressure

**Figure 1:**
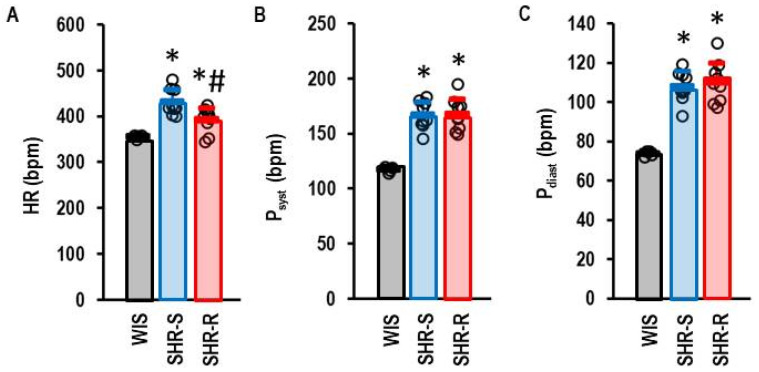
Effect of free voluntary wheel activity on heart rate and blood pressure. (**A**) Heart rate (HR) is given for normotensive Wistar rats (WIS; *n* = 4), spontaneously hypertensive rats held under standard (sedentary) conditions (SHR-S; *n* = 9), and spontaneously hypertensive rats with free access to running wheels (SHR-R; *n* = 9). (**B**) Systolic blood pressure (P_syst_) and (**C**) diastolic blood pressure (P_diast_) is given for the same rats. Data are given as means ± S.D. with original data points as dots. Statistical comparison was done by one-way ANOVA with *p* = 0.000225 for HR, *p* = 0.000004 for P_syst_, and *p* = 0.000002 for P_diast_ with Student–Newman–Keul’s post hoc analysis. *; *p* < 0.05 vs. WIS; #, *p* < 0.05 vs. SHR-S.

Intervention (access to running wheels) started at the age of six weeks. Rats were sacrificed six months later. Physical activity was quantified after two weeks with access to running wheels. SHRs with access to running wheels run 71.5 ± 25.9 km/week (range: 41.2–115.6 km/week) with a velocity of 2.39 ± 0.28 km/h (range: 1.86–2.79 km/h). On average, they spent 29.7 ± 9.4 h per week in the wheels (range: 16.4–44.1 h/week). Two weeks prior to scarification rats were trained to use the blood pressure apparatus and blood pressure was then investigated in the following week. SHRs had elevated heart rate and blood pressure compared with normotensive rats (Figure 1A–C). VRWA reduced resting heart rate in SHRs from 432 ± 27 bpm to 393 ± 26 bpm (effect size: 1.405; *p* = 0.009). However, neither the systolic nor the diastolic blood pressures were affected by VRWA. The data confirmed the known and expected training effects on the heart rate in this model.

### 2.2. Effect of Hypertension of Gene Expression in the Brain

**Figure 2:**
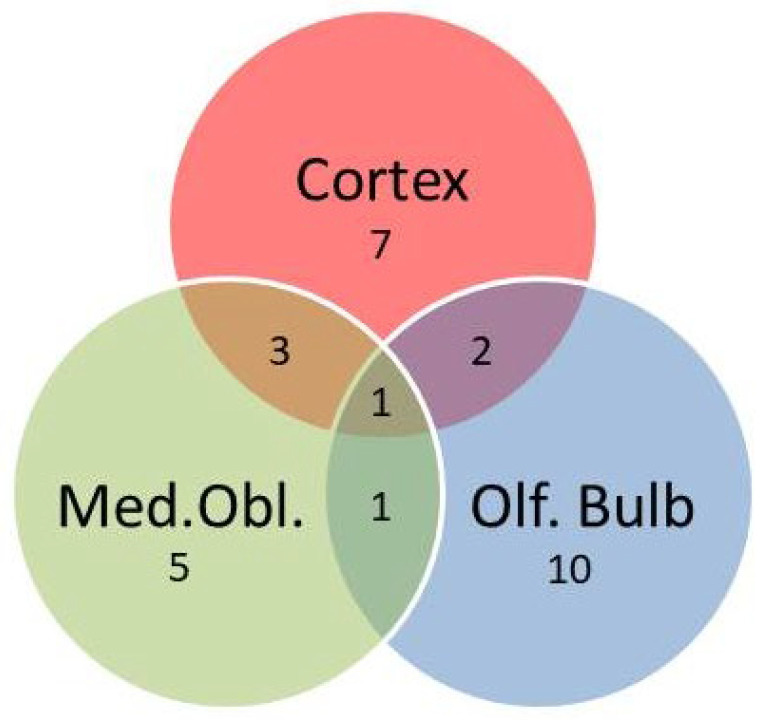
Venn diagram indicating the number of differentially regulated genes (DRGs) in the three brain areas that show a correlation with blood pressure. Of note, one gene (*Ucp2*) is differentially expressed in all three parts of the brain.

Next, twenty-five genes coding for proteins involved in control of oxidative stress, metabolism, inflammation, local perfusion, or linked to dementia when dysregulated were quantified in the three areas of the brain that were investigated here. As indicated in the Venn diagram (Figure 2), hypertension affected several of these marker genes.

**Figure 3:**
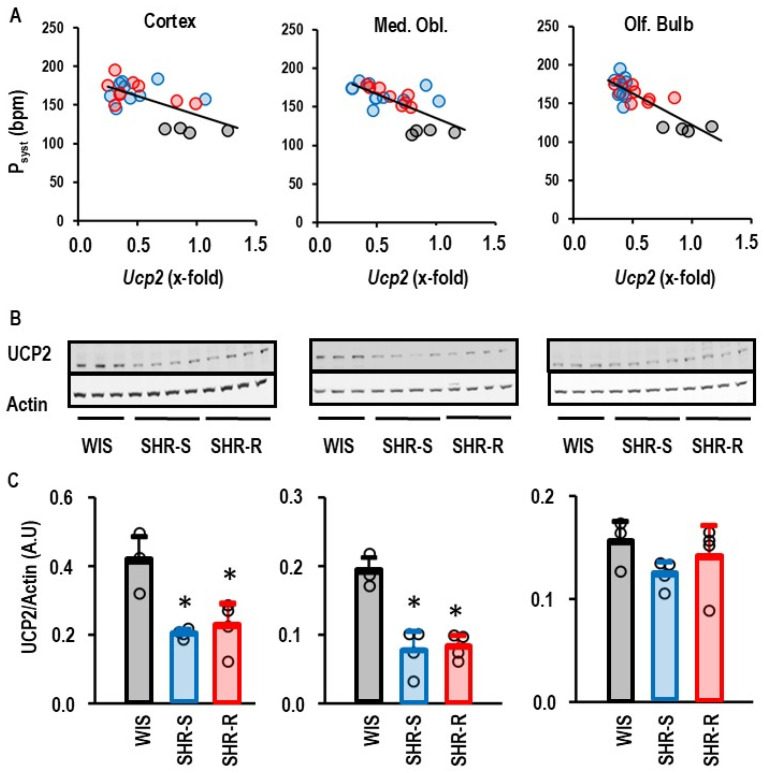
Correlation between blood pressure and *Ucp2* expression. (**A**) Black circles indicate data from WIS (*n* = 4), blue circles indicate data from SHR-S (*n* = 9), and red circles indicate data from SHR-R (*n* = 9). *p* values are 0.015 (cortex; r^2^ = 0.406), 0.001 (medulla oblongata; r^2^ = 0.440), and *p* < 0.000 for olfactory bulb (r^2^ = 0.669). (**B**) Western blots indicating the UCP2 protein expression in the different areas from the brain. (**C**) Quantification of the Western blots. Data are given as means ± S.D. with original data points as dots. WIS (*n* = 3); SHR-S (*n* = 4); SHR-R (*n* = 4) *, *p* < 0.05 vs. WIS.

Most genes that correlated with blood pressure were detected in the olfactory bulb (*Ucp2*, *Vegfa*, *Ucp5*, *Ace2*, *Ccl2*, *Cat*, *Psen1*, *Psen2*, *Rag*, *Bace1*), followed by the cortex (*Ucp2*, *Ucp4*, *Il6*, *Nppa*, *Vegfa*, *Ren*, *Sg2*), and the medulla oblongata (*Ucp2*, *Nppa*, *Ren*, *Cat*, *Upc3*). Most interestingly, we identified *Ucp2* as the only gene that is affected by hypertension in all three parts of the brain (Figure 3A). As expected from gene expression data, hypertensive rats also had less UCP2 protein expression (Figure 3B,C).

**Figure 4:**
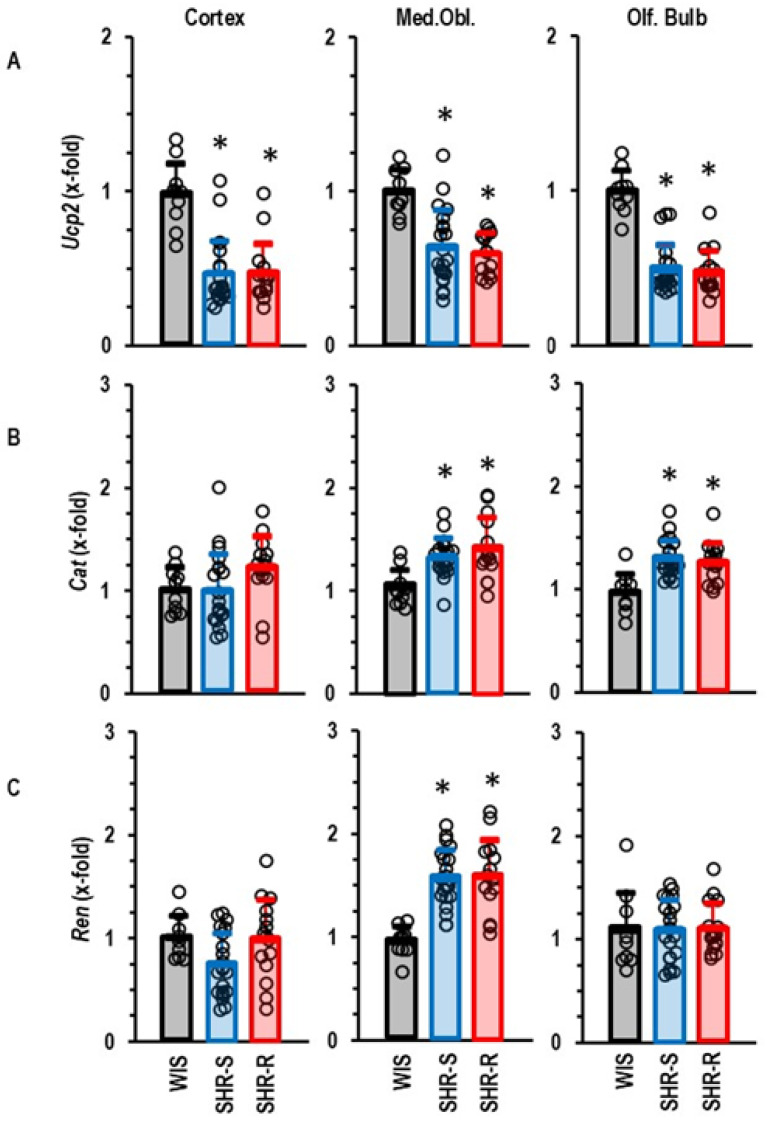
Group comparison of the gene expression of (**A**) *UCP2*, (**B**) *CAT*, and (**C**) *REN* in the three different parts of the brain. Data are means ± S.D. and the expression in WIS (*n* = 10) was set as 1.0 and compared with SHR-S (*n* = 21) and SHR-R (*n* = 15). Statistical comparison was done by one-way ANOVA with *p* values of *p* < 0.000 (*Ucp2*, cortex), *p* < 0.000 (*Ucp2*, med. obl.), *p* < 0.000 (*Ucp2*, olf. bulb), *p* = 0.001 (*Cat*, med. obl.), *p* < 0.000 (*Cat*, olf. bulb); and *p* < 0.000 (*Ren*, med. obl.). All other comparisons with *p* > 0.005. Student–Newman–Keul’s post hoc analysis was performed. Data are given as means ± S.D. with original data points as dots. *, *p* < 0.05 vs. WIS.

Downregulation of *Ucp2* in the brain may lead to oxidative stress but physical activity should, vice versa, reduce oxidative stress. However, VRWA did not affect the mRNA expression of *Ucp2* (Figure 4A). Nevertheless, the expression of *Cat* was slightly increased in hypertensive rats (Figure 4B). Furthermore, hypertension induced *Ren* expression in the medulla oblongata. Neither the effect of hypertension on the *Cat* expression nor that on the *Ren* was affected by VRWA (Figure 4B,C).

### 2.3. Effect of Physical Activity on the Expression of Genes in Female SHRs and Oxidative Stress

**Figure 5:**
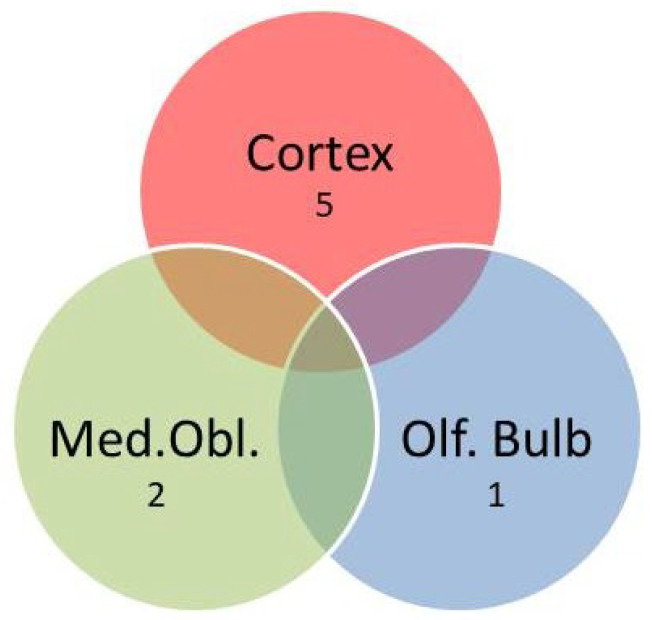
Venn diagram indicating the number of differentially regulated genes (DRGs) in the three brain areas that show a correlation with running activity (km/w). Of note, none of the genes were differentially expressed in all three parts of the brain.

The Venn diagram (Figure 5) shows that VRWA modified the gene expression mainly in the cortex (*Ucp3*, *Ace1*, *Vegfa*, *Rag*, *Nse*). In the medulla oblongata, *Slc2a4* and *Nppa* showed an inverse relationship between activity and expression, and in the olfactory bulb only *Ren*.

**Figure 6:**
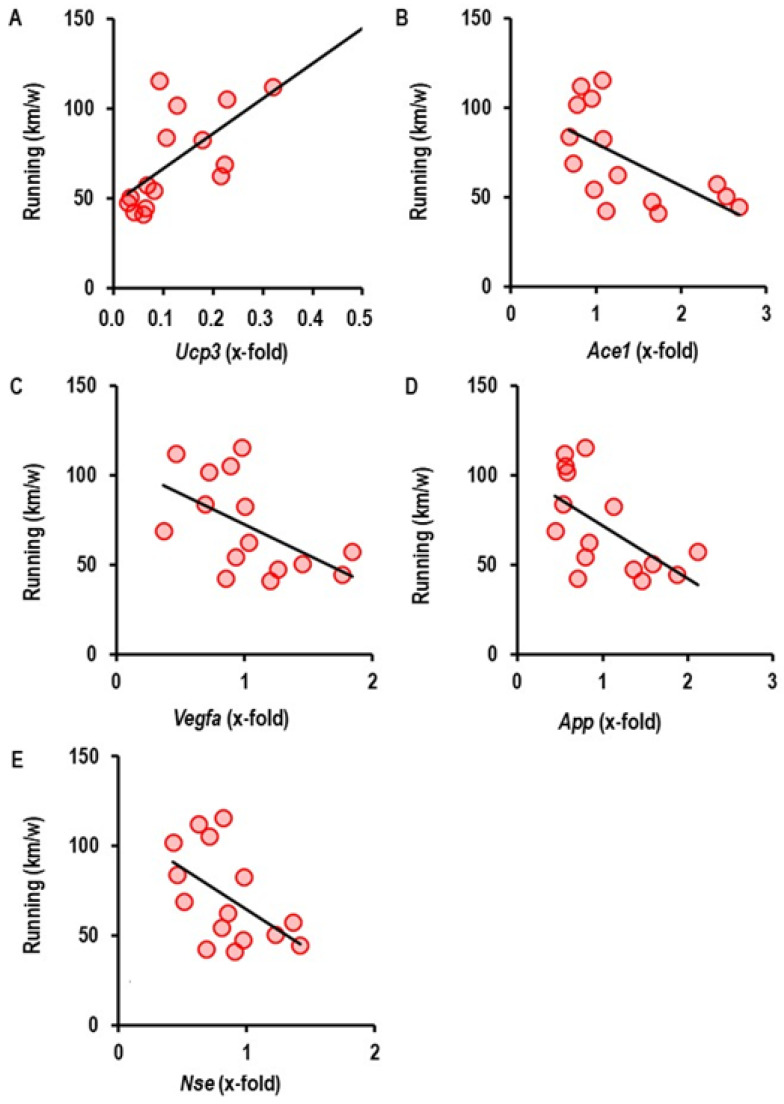
Correlation between running activity and gene expression in the cortex (*n* = 15). *p* values are 0.009 (**A**, *Ucp3*; r^2^ = 0.412), 0.016 (**B**, *Ace1*; r^2^ = 0.369), 0.036 (**C**, *Vegfa*; r^2^ = 0.295), 0.020 (**D**, *App*; r^2^ = 0.350), and 0.043 (**E**, *Nse*; r^2^ = 0.280).

In particular, the expression of *Ucp3* was upregulated in an activity-dependent way, whereas genes favoring oxidative stress such as *Agtr1* and those linked to vascular dementia such as *App* and *Nse* were downregulated. In addition, physical activity did affect the expression of *Vegfa* (Figure 6).

**Figure 7:**
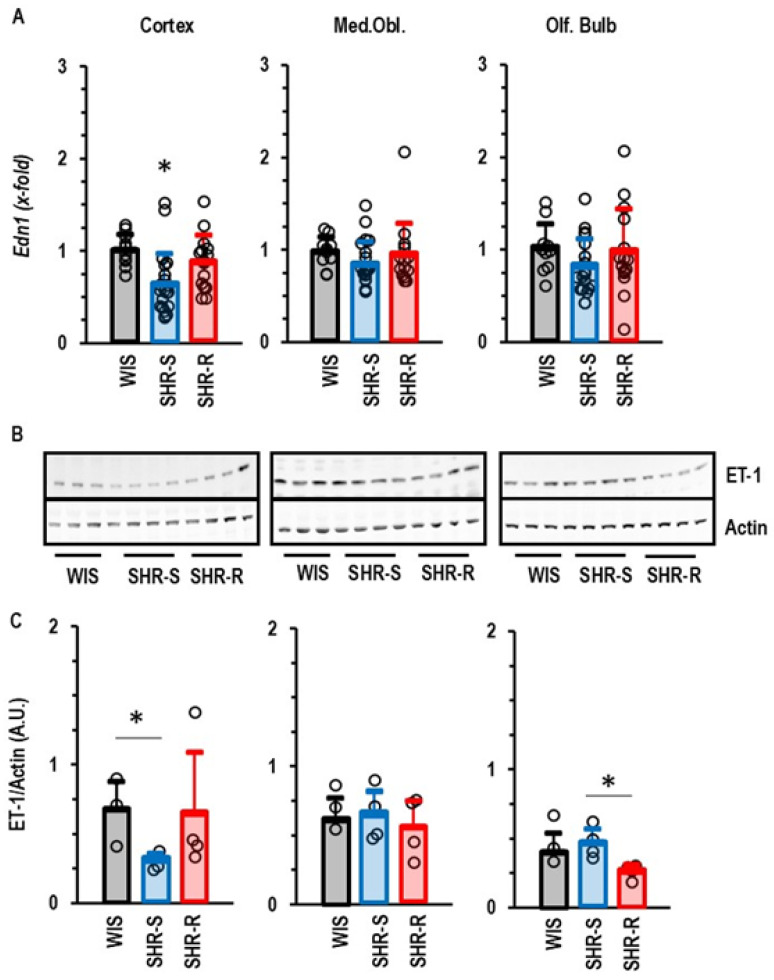
Group comparison of endothelin-1 expression in the three different parts of the brain. (**A**) Data are means ± S.D. and the expression in WIS (*n* = 10) was set as 1.0 and compared with SHR-S (*n* = 21) and SHR-R (*n* = 15). Statistical comparison was done by one-way ANOVA with *p* values of *p* < 0.05. (**B**) Western blots indicating the expression of endothelin-1. (**C**) Quantification of the Western blots (WIS, *n* = 3; SHR-S; *n* = 4; SHR-R, *n* = 4). Statistical comparison was done using a Kruskal–Wallis test with *p* values of 0.056 (cortex) and 0.069 (olf. bulb). *t* test differences with *p* < 0.05 are indicated by asterisks. Data are given as means ± S.D. with original data points as dots.

For the genes shown in Figure 7, we obtained a statistically validated correlation between exercise intensity and expression. In addition, cortical expression *End1* was also affected by exercise but no strong correlation between exercise intensity and expression occurred. Nevertheless, cortical expression of *End1* was reduced in SHR-S rats on the mRNA and protein level (Figure 7). However, this reduction was normalized to values observed in normotensive rats in the exercise-performing rats (Figure 7). In contrast, SHR-S rats did not show a differential expression of endothelin-1 in the other parts of the brain. VRWA slightly reduced endothelin-1 protein expression in the olfactory bulb in a post-translational regulated manner (Figure 7).

**Figure 8:**
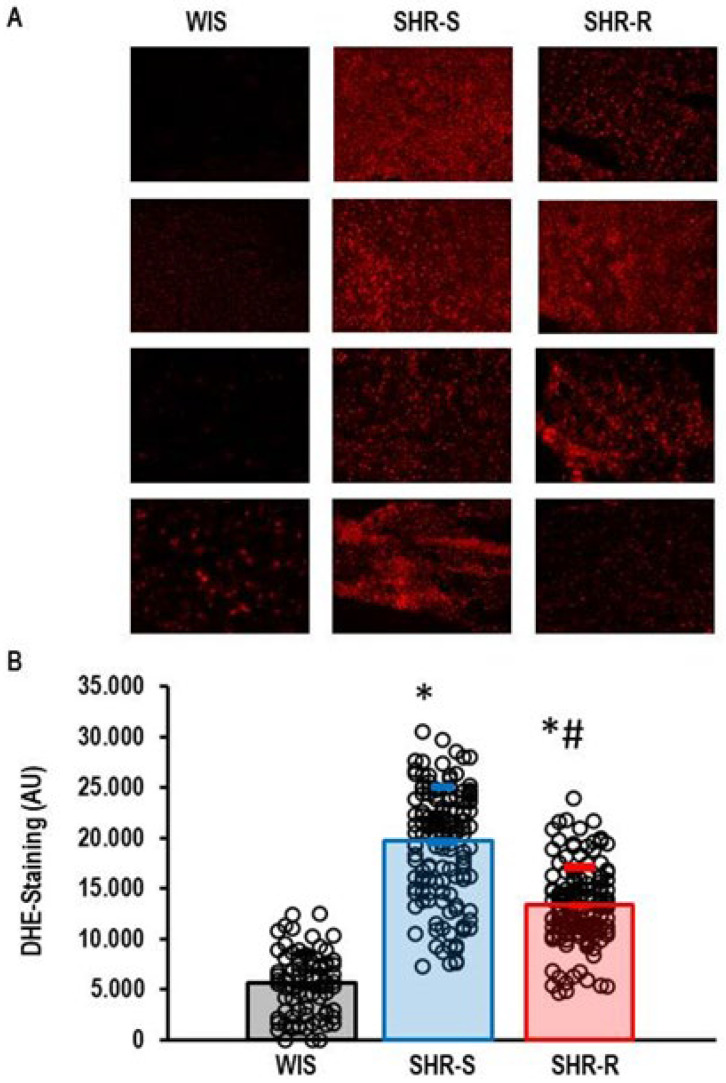
Visualization of oxidative stress by DHE staining. Cortical slices were stained. (**A**) Representative original images of four rats. (**B**) Means ± S.D. of these slices. Statistical comparison was done by one-way ANOVA with *p* values of *p* < 0.000. Student–Newman–Keul’s post hoc analysis was performed. *; *p* < 0.05 vs. WIS; #; *p* < 0.05 vs. SHR-S. Data are given as means ± S.D. with original data points as dots.

The downregulation of UCP2 in hypertensive rats may contribute to oxidative stress. We found that DHE staining, a marker of oxidative stress, was indeed increased in SHRs (Figure 8).

**Figure 9:**
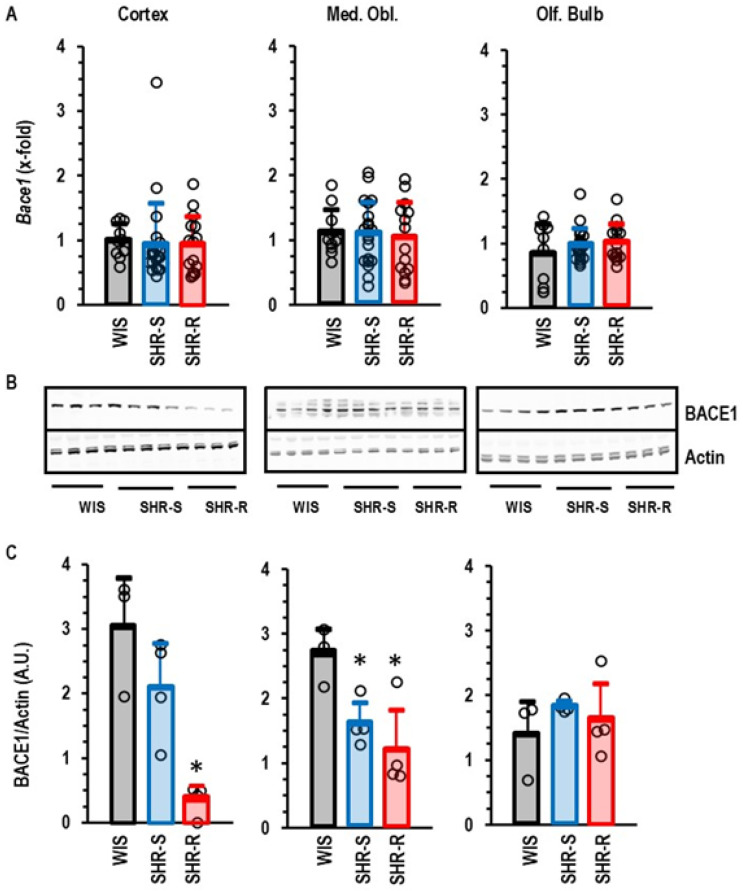
Group comparison of BACE1 expression in the three different parts of the brain. (**A**) Data are means ± S.D. and the expression in WIS (*n* = 10) was set as 1.0 and compared with SHR-S (*n* = 21) and SHR-R (*n* = 15). Statistical comparison was done by one-way ANOVA with *p* values of *p* > 0.05 for all groups. (**B**) Western blots indicating the expression of BACE1. (**C**) Quantification of the Western blots. Statistical comparison was done using one-way ANOVA with Student–Newman–Keuls post hoc analysis. *; *p* < 0.05 vs. WIS. Data are given as means ± S.D. with original data points as dots.

Finally, we found an activity-dependent downregulation of *App* and *Nse*. The expression of both genes is linked to dementia. Furthermore, although neither hypertension nor voluntary running wheel activity changed the mRNA expression of *Bace1*, the protein expression of this secretase was reduced in the cortex of SHRs performing VRWA activity (Figure 9).

## 3. Discussion

Oxidative stress plays a major role in concepts that explain how vascular defects caused by hypertension increase the risk of dementia [18]. In this context, a new finding of our study is that hypertension downregulates the expression of *Ucp2* in various areas of the brain. UCP2 is a neuroprotective molecule and upregulation of UCP2 can decrease oxidative stress, whereas its downregulation induces oxidative stress in neurons [22,28,29,30,31,32,33]. Downregulation of UCP2 as part of mitochondrial dysfunction has been linked to Alzheimer’s disease [22,33,34]. Our study shows that hypertension downregulates *Ucp2* expression in all three parts of the brain that were under investigation in this study. Furthermore, we confirmed downregulation of UCP2 on the protein level in the cortex and medulla oblongata, whereas the downregulation of the *Ucp2* gene expression in the olfactory bulb was not accompanied by a similar downregulation on the protein level. This finding shows that post-translational modifications can differentially affect the regulation of UCP2 protein expression in various parts of the brain. We investigated whether life-long high physical activity can ameliorate the hypertension-dependent downregulation of *Ucp2* gene expression in the brain. That was, however, not the case. Furthermore, although the downregulation of *Ucp2* gene and protein expression in the cortex was associated with increased oxidative stress, as predicted by some publications, rats with VRWA showed less oxidative stress than sedentary SHRs but no improvement in *Ucp2* expression. Therefore, we do not believe that the main function of UCP2 in the brain is its anti-oxidative function. However, we found an increased expression of *Ucp3* and a downregulation of *Ace1* in the cortex of hypertensive rats with VRWA. Both effects are in line with our observation of less oxidative stress.

UCP2 is commonly expressed in all parts of the brain and is expressed in a much stronger way than in UCP3 [35,36]. A compensatory upregulation of UCP isoforms in situations in which UCP2 is downregulated has been described before; however, in the brain this was mainly the case for UCP4, with UCP3 not considered as a UCP isoform present in the central nervous system. In contrast to these assumptions, which were mainly generated in mice, we found a small but stable signal for *UCP3* expression in all areas under investigation of the rat brain. More importantly we observed the highest expression in hypertensive rats in those animals with the highest running activity. This may indicate that, at least in part, *Ucp3* can also be upregulated in the rat brain. In contrast, neither *Ucp4* nor *Ucp5* were affected by physical activity and were less affected by blood pressure. This indicates a completely different role for the atypical brain-specific isoforms UCP4 and UCP5 than for UCP2 and UCP3, a finding consistent with the suggestions that UCP4 and UCP5 are rather different from the classical UCP isoforms [37].

A very likely explanation of how improved physical activity reduces vascular stress and thereby dementia is to suggest that exercise lowers the blood pressure and thereby reduces the mechanical load on vessels. We have shown before, in a number of studies, that VRWA in SHRs does not reduce blood pressure. In accordance, no such effect was observed in this new study. In addition to our previous findings, we now show a rationale for this behavior. We found an upregulation of *Ren* in the medulla oblongata that was not affected by VRWA. This observation is important, as SHR is a low-renin model when analyzing renin expression in the kidney [38,39]. Collectively, the data show that VRWA does not reduce blood pressure and therefore does not reduce the mechanical load to vessels. The effects of VRWA on altered cortical expression must have another reason. As such, we identified the effect of VRWA on the protein expression of endothelin-1 that was downregulated in the cortex of SHRs but normalized in SHRs with VRWA. Downregulation of endothelin-1 expression in the brain has been reported before under basal conditions and exposure to smoke [40,41]. Binding affinity of endothelin-1 to receptors is indistinguishable between SHR and normotensive Wistar rats [42]. Therefore, the altered expression of endothelin-1 in the cortex of SHR and its normalization by physical activity may have functional relevance. The bulbus olfactorius is involved in the regulation of blood pressure in rats. The downregulation of endothelin-1 by high physical activity in this part of the brain was, however, unable to normalize the blood pressure [43,44].

Finally, we investigated whether hypertension and physical activity can modify the gene and protein expressions of molecules that are directly associated with dementia. We found that VRWA is inversely associated with the gene expression of *App* and *Nse*, two proteins linked to Alzheimer’s disease [45,46]. Furthermore, we found a post-transcriptional modification of BACE1 protein expression by VRWA in the cortex. This finding is in line with an earlier report that exercise can reduce the BACE1 expression after administration of Aβ particles. The question of whether the observed effects of high physical activity on these proteins are linked to the lowering of oxidative stress needs further study. Such studies must lower oxidative stress in the cortex to a comparable amount to that seen by VRWA, though using a direct anti-oxidative effect that is independent from physical activity. However, it is already known that inhibition of the renin–angiotensin pathway can inhibit the induction of BACE1 and that hyperactivity of the renin–angiotensin system worsens cognitive function [47]. Our finding of an activity-dependent effect on *Ace1* (reducing renin-pathway activity) and lowering of BACE1 expression is in line with these findings, though the molecular mechanisms in how these effects may be linked are not well understood.

## 4. Materials and Methods

The investigations agree with the “Guide for the Care and Use of Laboratory Animals” purchased by the U.S. National Institute of Health (NIH Publication No. 85-23, revised 1996). The study was approved by the local authorities (RP Gießen; V 54-19 c 20 15 h 01 GI 20/1 Nr. 76 and GI 20/1 Nr. 77/2014).

Animal Model: Forty-six six-week-old female spontaneously hypertensive rats were randomly selected in either a sedentary control group under normal holding conditions (SHR-S; *n* = 21) or exposed to improved holding conditions with free access to running wheels during their active nighttime (SHR-R; *n* = 15). Age-matched normotensive Wistar rats served as controls (WIS; *n* = 10).

Rats were kept with two rats per cage during the light period, and rats with VRWA were set individually in cages with running wheels during the night period. Cage size was L: 595 mm, B: 380 mm; H: 200 mm (in cages with running wheels 270 mm). Temperature was held constant at 21 ± 1 °C, humidity 60%, and day–night cycle 12:12 h. Rats were fed ad libitum with Harlan Teklad Global 18% Protein Rodent Diet. Running wheels were connected to a bike computer that calculated the number of rotations and time in use and which could be translated into km by quantification of the circumference of the wheels. Rats were purchased from Harlan Laboratories Bioservice GmbH; Walrode, Germany.

Here, we used exclusively female rats for the following reasons: First, female rats display a higher voluntary running wheel activity compared with male rats and therefore allow a better analysis of the effect of running. Second, female rats display an age-dependent degree of mitochondrial number to the proteins that are the focus of this study, though the quality of mitochondria is improved by upregulation of UCP4 and UCP5 [48]. Third, restriction to one sex reduces variability in experimental data and therefore allows use of fewer animals.

**Figure 10:**
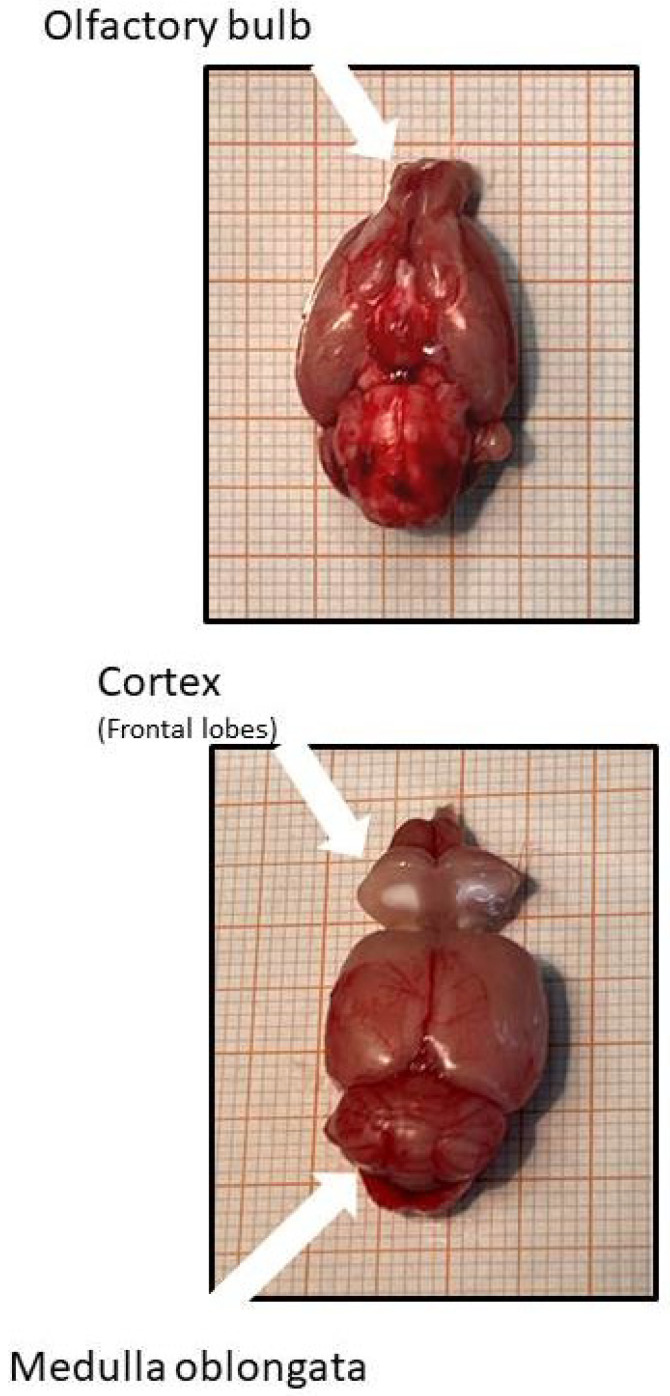
Brain preparation from rats showing the position of the tissues used in this study.

After 7.5 months, rats were anesthetized by isoflurane inhalation. After cervical dislocation, brains were prepared and the cortex, medulla oblongata, and olfactory bulb were extracted and immediately transferred to fluid nitrogen and stored at −80 °C until use (Figure 10).

Analysis of physiological parameters: Rats started to use running wheels at the age of six weeks, thus prior to the onset of hypertension in SHRs. Running wheels were connected to a computer and the duration of wheel use was recorded as well as the total distance. From these data we calculated the run performance (expressed as km per week) for each rat of the running groups and the average speed (km/h).

Two weeks prior to the end of the experiments some rats from each group were randomly selected and the blood pressure and heart rate were analyzed using a tail-cuff method as described before [49].

RT-PCR: Total RNA was isolated from brains using peqGOLD TriFast according to the manufacturer’s protocol. Genomic DNA was removed by treatment of samples with 1 U DNase/µg RNA for 15 min at 37 °C. One microgram of RNA was used in a 10 µL reaction to synthesize cDNA using Superscript RNase H Reverse Transcriptase and oligo (dt) as primers. The sequences of the primers used are summarized in Supplementary Table S1. Primers used in this study covered the following pathways: Oxidative stress (*Sod2*, *Cat*), metabolism (*Slc2a1*, *Slc2a4*), inflammation (*Ccl2*, *Il6*), perfusion (*Agtr1*, *Agtr2*, *Ace1*, *Ace2*, *Ren*, *End1*, *Endrb*, *Ece1*, *Nppa*), uncoupling proteins (*Ucp2*, *Ucp3*, *Slc25a27*, *Slc25a14*), and Alzheimer’s disease (*Psen1*, *Psen2*, *Sg2*, *App*, *Bace1*, *Nse*, *Rag*). Expression was normalized to *B2m*. Quantification was based on the ΔΔC_T_ method and performed as described before [50].

Western Blot: Total protein was extracted from the cortex, medulla oblongata, and olfactory bulb using cell lysis buffer (10×) (Cell Signaling Technology, Frankfurt, Germany) according to the manufacturer’s protocol. Briefly, the homogenate was centrifuged at 14,000× *g* at 4 °C for 10 min and the supernatant was treated with Laemmli buffer (Sigma-Aldrich, Taufkirchen, Germany). The protein concentration was adjusted to 20 µg/µL. Recombinant hUCP-2 (kindly provided by E. Pohl, University of Veterinary Medicine, Vienna, Austria) was used as a positive control. Protein samples were loaded on NuPAGE Bis-Tris Precast gels (10% for UCP-2 and BACE1, 12% for endothelin-1; Life Technology Darmstadt, Germany) and, subsequently, transferred to nitrocellulose membranes. The expression of UCP-2 was analyzed with an antibody (also kindly provided by E. Pohl), whose specificity has been evaluated before [51,52]. The expression of endothelin-1 was analyzed with an antibody purchased from MyBiosource, San Diego, CA, USA (product MBS2540132), and BACE1 was analyzed with an antibody purchased from GeneTex, Hsinchu, Taiwan (product GTX134480). Expression of all proteins was normalized to the expression of Actine, using an antibody produced in rabbit from Cell Signaling, Danvers, MA, USA (product 4968). Secondary antibodies (horseradish peroxidase-coupled secondary antibody) directed against rabbit IgG was purchased from Dako (now Agilent Technologies, Santa Clara, CA, USA).

Dihydrethidium (DHE) staining: To perform DHE staining, cryosections of every part of the samples (the cortex, medulla oblongata, and olfactory bulb) were incubated with dihydroethidium (DHE, D23107, Thermo Fisher Scientific, Dreieich, Germany) dissolved in 1 X PBS for 10 min at 37 °C in a light-protected humidity chamber, then fixed with Dako Fluorescent Mounting Medium (Dako, North America Inc., Santa Clara, CA 95051, USA, USA). Slides were imaged by Keyence microscope (BZ-X800 Keyence, Neu-Isenburg, Germany). Using an excitation wavelength of 545 nm, with emission recorded at 605 nm. The mean fluorescence intensity of *n* = 4 preparations was used to quantify the extent of superoxide.

Statistics: Data are expressed as means ± S.D. or presented as size effects with 5% and 95% confidence intervals. *p* values were calculated by ANOVA with Student–Newman–Keuls post-hoc analysis. Effect Sizes were analyzed by Cohen’s d. SPSS 27 was used to calculate all the data.

## 5. Conclusions

Our study shows that hypertension leads to a strong downregulation of the neuro-protective mitochondrial uncoupling protein. Furthermore, we show that VRWA, as a surrogate parameter of an active lifestyle, can reduce hypertension-dependent modifications in gene and protein expressions in the cortex. As the cortex is important for cognition and memory, such effects are important for the long-term prognosis of patients. Here, we observe that the expressions of genes coding for two proteins that potentially favor oxidative stress (*Ucp3*, *Ace1*) are downregulated in the cortex of hypertensive rats with high physical activity. Furthermore, the expression of genes associated with Alzheimer’s disease are also affected (*App*, *Nse*). Furthermore, downregulation of *Vegfa* may indicate better oxygenation of the brain. Therefore, our study suggests a mechanistic hint on how physical inactivity in hypertensive patients increases the risk of developing dementia.

## Data Availability

The original contributions presented in this study are included in the article/Supplementary Materials. Further inquiries can be directed to the corresponding author.

## Supplementary Materials

The following supporting information can be downloaded at https://www.mdpi.com/article/10.3390/ijms27073182/s1.

## Abbreviations

The following abbreviations are used in this manuscript:

UCP	Uncoupling protein
VRWA	Voluntary running wheel activity
