# Supplementary material for: Effects of Voluntary Running Wheel Activity and Hypertension on the Brain of Female Spontaneously Hypertensive Rats (SHRs)
Source: Int J Mol Sci. 2026 Mar 31;27(7):3182. doi: 10.3390/ijms27073182 (PMC13072940; doi:10.3390/ijms27073182)
Supplement: Supplementary file 1 [file ijms-27-03182-s001.zip › ijms-4194959-supplementary.pdf]

Supplement Table S1: List of Primers used in the study

Name	forward	reverse	NCBI
Ace1	ACAGCTATAACTCGAGTGCCG	ACAGCTCCTTGGCCTTCTTG	NM_012544.1
Ace2	ACAATTGTTGGAACGCTGCC	CGCTTCATCTCCCACCACTT	NM_001012006.2
Agtr1	GAAGTCTCGCCTTCGCCGCA	CAGCCCTATGGGGAGCGTCG	NM_030985.4
B2m	GCCGTCGTGCTTGCCATTC	CTGAGGTGGGTGGAACTGAGAC	NM_012512.2
Bace1	CCACAGACGCTCAACATCCT	CCATGAGGGATGCTCACCAG	NM_019204.2
Cat	TTTTCACCGACGAGATGGCA	CTGACTCTCCAGCGACTGTG	NM_012520.2
Ccl2	TCACGCTTCTGGGCCTGTTGT	TCCAGCCGACTCATTGGGATCA	NM_031530.1
Ece1	TCTGGCCAACATCACCATCC	TAGACCACGATGGGCTCAGA	NM_053596.2
Edn1	CCGTATGGACTAGGAAGCCC	TGCATGGTACTTTGGGCTCG	NM_012548.2
Ednrb	GCTAGCCATCACTGCGATCT	TGTCTTGGCCACTTCTCGTC	NM_017333.1
Hif2a	GGGTACGTGAGGCATGTTGA	CCGTCGGTCAGACCAGAAAA	NM_024359.1
Il6	CACTTCACAAGTCGGAGGCT	TCTGACAGTGCATCATCGCT	NM_012589.2
Nppa	ATGGGCTCCTTCTCCATCAC	TCTTCGGTACCGGAAGCTG	NM_012612
Nse	GGAACAAGTTGGCCATGCAG	TCTCCAGGATATTGGGGGCA	AF019973.1
Psen1	ATGCTGGTCGAAACAGCTCA	GGTACCCTCCTTTGGGCTTC	NM_019163.4
Psen2	ACTCCGTGCTGAACACTCTG	AGGAGCATCAGGGAGGACAT	NM_031087.2
Rag	TAAGCGATGCCCTTCTCGTG	CTGCATGTCTCTTTGGCAACA	X07648.1
Ren	GAGGCAGTGACCCTCAACAT	GTGTCCACCACTGCCATACA	NM_022177.3
Sg2	ATACACCAGGCTCAACAGGC	ATCTCCTTGCACACTGTCGG	X13231.1
Slc2a1	GCTGTGGCTGGCTTCTCTAA	CCGGAAGCGATCTCATCGAA	NM_138827.1
Slc2a4	ACCGTCTTCACGTTGGTCTC	ATCAAGATGGCACAGCCACA	NM_012751.2
Slc25a14	GCAGCAGTGATTGTAAGCGG	ACAGGGAAAGTGCCGAACTC	NM_053501.3
Slc25a27	GCCGATGTCATCAAAAGCCG	CCCTGCAAATCGCAGTCTCAT	XM_006247484.3
Sod2	ATGTTGTGTCGGGCGGCGTG	TCGCGTGGTGCTTGCTGTGG	NM_017051
Ucp2	CACCGTCATTGCCTCCCCCG	CGGAGCATGGTCAGGGCACA	NM_019354.2
Ucp3	GACCCACGGCCTTCTACAAA	TCAAAACGGAGATTCCCGCA	NM_009464.3
Vegfa	TGCCCCTAATGCGGTGTGCG	GGCTCACAGTGAACGCTCCAGG	NM_001171624.2
